# Social Networking Site Use While Driving: ADHD and the Mediating Roles of Stress, Self-Esteem and Craving

**DOI:** 10.3389/fpsyg.2016.00455

**Published:** 2016-03-30

**Authors:** Ofir Turel, Antoine Bechara

**Affiliations:** ^1^Information Systems and Decision Sciences, California State University, FullertonFullerton, CA, USA; ^2^Psychology, Brain and Creativity Institute, University of Southern CaliforniaLos Angeles, CA, USA

**Keywords:** facebook use, ADHD, addiction and addiction behaviors, cravings, self esteem, social networking sites

## Abstract

**Background:** Adults who present ADHD symptoms have an increased risk for vehicle accidents. One conceivable overlooked account for this association is the possibility that people with ADHD symptoms use rewarding technologies such as social networking sites (SNS) while driving, more than others. The objective of this study was to understand if and how ADHD symptoms can promote SNS use while driving and specifically to conceptualize and examine mechanisms which may underlie this association. To do so, ADHD is viewed in this study as an underlying syndrome that promotes SNS use while driving in a manner similar to how addictive syndromes promote compulsive seeking of drug rewards.

**Methods:** Time-lagged survey data regarding ADHD, stress, self-esteem, SNS craving experience, SNS use while driving, and control variables were collected from a sample of 457 participants who use a popular SNS (Facebook) and drive, after face-validity examination with a panel of five users and pretest with a sample of 47. These data were subjected to structural equation modeling (SEM) analyses using the frequency of ADHD symptoms measured with ASRS v1.1 Part A as a continuous variable, as well as multivariate analysis of variance using ADHD classification based on ASRS v1.1 scoring guidelines.

**Results:** ADHD symptoms promoted increased stress and reduced self-esteem, which in turn, together with ADHD symptoms, increased one's cravings to use the SNS. These cravings ultimately translated into increased SNS use while driving. Using the ASRS v1.1 classification, people having symptoms highly consistent with ADHD presented elevated levels of stress, cravings to use the SNS, and SNS use while driving, as well as decreased levels of self-esteem. Cravings to use the SNS among men were more potent than among women.

**Conclusion:** SNS use while driving may be more prevalent than previously assumed and may be indirectly associated with ADHD symptoms. It is a new form of impulsive and risky behavior which is more common among people with symptoms compatible with ADHD than among others. Consistent with addiction and decision making models, SNS use while driving can be viewed as a form of a compensatory reward seeking behavior. As such, prevention and reduction interventions that target the mediating perceptions and states should be devised.

“If 24% of drivers aged 17–24 were driving around drunk, there would be a massive public outcry. This [using smartphones for email and social networking while driving] is much worse, but we blindly accept this clash of technologies which is costing thousands of lives” (Hanlon, 2012).

## Introduction

Attention deficit/Hyperactivity disorder (ADHD) is a neurodevelopmental psychiatric impairment which normally develops before the age of 7 years; it manifests through symptoms involving high distractibility, poor sustained attention, and high impulsiveness-hyperactivity (Jensen et al., 1997). The etiology and pathogenesis of this disorder are broad, and include functional abnormalities in brain structures associated with decision-making. These may include structures such as the striatum and its neurotransmitter dopamine, which is linked to increased impulsivity (Lou, 1996), and the prefrontal cortex, which when impaired, leads to reduced inhibition abilities (Zametkin and Liotta, 1998). These neuro-behavioral deficiencies may be associated primarily with genetics, but also with “nurture” factors such as upbringing and socio-economic status (Cortese, 2012).

Recent studies have shifted attention to the fact that ADHD can persist or merely be observed during adulthood (Davidson, 2008), and that adults too can often present a range of ADHD symptoms (Fayyad et al., 2007). It is estimated that about 4.4% (Kessler et al., 2006) to 5.2% (Fayyad et al., 2007) of the US population meets strict ADHD classification criteria, and many others suffer from ADHD-associated symptoms and are not diagnosed. ADHD symptoms in adults have been linked to mood and anxiety problems, risky behaviors such as substance abuse (Kessler et al., 2006), overeating and obesity (Davis et al., 2006), reduced cognition, and problems in social interactions (Fayyad et al., 2007). This range of outcomes puts a heavy burden on people with symptoms compatible with ADHD, which further drives decreased sleep quality, increased hospital visits and stays, and reduced subjective health and wellbeing among them (Kirino et al., 2015).

Adults with ADHD can also be between 1.5 (Chang et al., 2014) to almost four (Barkley et al., 1993) times more likely than others to be involved in vehicle accidents. This presumably happens due to, among other reasons, inattention to the road (Barkley and Cox, 2007; Cox et al., 2011). One possible overlooked and contemporary explanation for this association, though, is the possibility that people with ADHD symptoms engage in using rewarding modern technologies such as Social Networking Sites (SNS) on their mobile devices while driving, more than others, even though this activity is dangerous and largely illegal and prohibited, at least in the United States of America. In essence, it is possible that modern technologies provide people with ADHD symptoms an incentive reward that elicits SNS use, even in risky situations such as when driving (Winstanley et al., 2006). The use of SNS can be highly rewarding and produce strong incentive rewards (Oh and Syn, 2015), more so for people with personality, self-esteem and social deficiencies (Sheldon et al., 2011), and perhaps even more so under stress conditions (Goeders, 2002; Aston-Jones and Harris, 2004). It is hence not surprising to find comorbidities between problematic and excessive use of technologies, negative and stressful states, and ADHD (Yoo et al., 2004; Yen et al., 2007). Nevertheless, possible associations between ADHD and the use of SNS while driving are yet to be explored.

A focus on the use of SNS while driving is worthy given the magnitude and prevalence of the potential harms of this behavior. For instance, at least 23% of car collisions involve cellphone use; and texting (including using SNS) while driving makes accidents 23 times more likely (TextingThumbBands.com, 2015). In addition, SNS use while driving is a major distraction which requires much attention; drivers' response time while using SNS such as Facebook was slowed by about 38% and the use of SNS while driving is consequently more dangerous than drinking, texting or driving under marijuana influence (Hanlon, 2012). Many drivers (about 27% in the US (Burns, 2015)), though, ignore such health and legal risks and use SNS while driving (RAC, 2011). Could ADHD symptoms be a culprit?

The objective of this study is to understand if and how ADHD symptoms can promote SNS use while driving, and specifically to conceptualize and examine mechanisms which may underlie this association. To do so, we rely on two perspectives borrowed from addiction and decision making research: the drive reduction theory of motivation and addiction (Wolpe, 1950; Brown, 1955), and the incentive motivational and psychostimulant perspective (Noel et al., 2013), both of which explain why people repeatedly engage in problematic behaviors. Borrowing from addiction and decision making models to explain behaviors under ADHD conditions is reasonable (Malloy-Diniz et al., 2007), since the underlying brain deficiencies of ADHD and addictions are similar and are associated with flawed incentive-reward and inhibition processes (Durston et al., 2003; Casey et al., 2007) and specifically with hypo-activity of brain systems involved in inhibition (Zametkin and Liotta, 1998) as well as hyperactivity of structures comprising what is known as the impulsive brain system (Lou, 1996).

From a drive reduction theory perspective, driving a car can be boring, deprive people from receiving internal rewards by using their SNS, and increase their concerns regarding what they may have been missing with their social contacts (Gil et al., 2015). Under these circumstances, people may develop strong and intrusive craving to use the SNS, which is unpleasant, and which may persist especially when driving (Collins and Lapp, 1992). These cravings motivate action, e.g., SNS use while driving, in order to eliminate the unpleasant cravings. The cravings may be stronger, more intrusive and involve more vivid imagery among people who suffer from a large cluster of ADHD symptoms, because these symptoms reduce people's ability to divert their attention from intrusive thoughts (Malloy-Diniz et al., 2007) and the use of SNS can be very rewarding for such individuals; children and adolescents with ADHD symptoms present hyper-responsiveness to social rewards (Kohls et al., 2009) which are often provided by SNS. This presumably happens because the use of the SNS can help such individuals present themselves in a more positive light (Gil-Or et al., 2015), escape their daily sorrows (Masur et al., 2014), increase their self-esteem and sociability (Zywica and Danowski, 2008), and reduce their loneliness (Deters and Mehl, 2013). Since the presence of ADHD symptoms often induces stress (Randazzo et al., 2008; Hirvikoski et al., 2009) and reduces people's self-esteem (Bussing et al., 2000; Richman et al., 2010), it is reasonable to assume that the magnitude of cravings to use the SNS is at least in part influenced by such aversive psychological states which result, at least in part, from having ADHD symptoms.

From the incentive motivational and psychostimulant perspective, ADHD is associated with reduced anticipation of rewards which promotes higher levels of reward-seeking behaviors (Scheres et al., 2007), occasionally with reduced frontal-striatal based inhibition (Nigg, 2005), and problems with delaying gratifications (Luman et al., 2005). All of these may be associated with increased cravings to use an SNS (Ko et al., 2009, 2013), even when driving, and ultimately engaging in risky use of SNS (Malloy-Diniz et al., 2007). Based on this perspective, the experience of cravings is a key driver of impulsive behaviors (Verdejo-Garcia and Bechara, 2009), which can be exacerbated by insular-cortex activity that promotes interoceptive awareness of such cravings, increases the reliance on mesolimbic dopamine systems (i.e., promotes impulsive behaviors), and diminishes one's ability to control such cravings (i.e., hypo-activation of prefrontal cortex systems; Naqvi et al., 2007; Naqvi and Bechara, 2010; Noel et al., 2013). Increased insular activity can be associated with the interoceptive awareness of the burdens which ADHD symptoms drive, such as increased stress (Flynn et al., 1999; Wright et al., 2003) and social pains in the form of reduced self-esteem (Eisenberger et al., 2011; Eisenberger, 2012; Hughes and Beer, 2013). Hence, from this perspective too, ADHD symptoms and their associated burdens (reduced self-esteem and increased stress) can promote reward-seeking behaviors and reduce one's ability to inhibit them (Noel et al., 2013).

Taken together, we propose to test the following hypotheses:

**H1a:** The level of ADHD symptoms will be positively associated with stress.
**H1b:** The level of ADHD symptoms will be negatively associated with self-esteem.
**H2a:** Stress will be positively associated with craving to use the Social Networking Site.
**H2b:** Self-esteem will be negatively associated with craving to use the Social Networking Site.
**H2c:** The level of ADHD symptoms will be positively associated with craving to use the Social Networking Site.
**H3:** Craving to use the Social Networking Site will be positively associated with Social Networking Site use while driving.

## Methods

### Study participants and procedures

All participants were students at a large North American university who have used a popular SNS, namely Facebook, at the time of the study, have been driving for school or work, and were not taking classes from the researchers. All participants signed informed consent forms (approved by the IRB of California State University, Fullerton) before completing the online surveys, and were given bonus points in their courses in exchange for their time. We began with a panel of five SNS users for face-validity examination, followed by a pilot study of 47 participants (out of 60, response rate of 78%) for scale pre-testing and validation. The pilot survey captured additional conceptually related measures (urge to use the SNS and the Temptation and Restraint inventory applied to SNS) as a means to establish internal validity, as well as self-reports of one's extent of SNS use as a way to establish predictive validity.

Time-lagged data for testing the model were then collected from a sample of 457 participants (out of 560, response rate of 82%) from the same university, using the same exclusion-inclusion criteria used in the pilot study. Data from this sample were collected at two points in time, one week apart, using online surveys posted on the class website. ADHD, self-esteem and control variables (age, sex, years on the SNS, number of SNS friends, social desirability, and SNS use habit) were measured at week 1. Stress, craving and SNS use while driving experienced after the first wave of data collection (“over the last week”) were captured in the second wave, at week 2. The time-lag design was employed to increase support in causality arguments and for reducing potential common method bias. Sample characteristics are outlined in Table 1. Examination of the frequencies of SNS use while driving revealed that 59.3% reported on never, or very rarely, doing so in the last week. Slightly over 40% of the sample reported on some level of use of the target SNS while driving in the previous week, and 5.5% reported on more than “often” engaging in this behavior.

**Table 1:** **Sample Characteristics**.

**Sample Attribute**	**Pilot Sample**	**Study Sample**
Size	*n* = 47	*n* = 457
Age	24.4 (4.10, 20–38)	23.4 (4.18, 18–60)
Sex Distribution (males/females)	24/23	225/232
Years of Experience on target SNS	4.07 (2.27, 0.6–10)	4.18 (2.23, 0.2–10)
Number of Friends on target SNS	311.62 (282.8, 20–1200)	339.87 (441.98, 1–4700)

### Instruments

The pilot study (*n* = 47) measured craving to use the target SNS, Facebook, using the Facebook Craving Experience (FaCE) scale which is an adaptation of the Alcohol Craving Experience (ACE) questionnaire (Statham et al., 2011) to the context of SNS which specifically focuses on one SNS, Facebook. The scale performed well in the pilot study with subscales presenting Cronbach's alphas between 0.85 and 0.94. The FaCE score was calculated by multiplying the average of the three (imagery, intensity and intrusion) frequency (FaCE-F) and strength (FaCE-S) scores of Facebook desire-related thoughts in the last week, as per the procedure described in Statham et al. (2011). Content validity was further established by correlating this score with a measure of urge to use Facebook (α = 0.90, *r* = 0.54, *p* < 0.001) adapted from Raylu and Oei (2004) and the Temptation and Restraint Inventory's (Collins and Lapp, 1992) second order factors applied to the current context, namely cognitive-emotional preoccupation with Facebook (α = 0.86, *r* = 0.60, *p* < 0.01) and cognitive-behavioral control efforts regarding Facebook use (α = 0.86, *r* = 0.42, *p* < 0.01). Predictive validity was established through association with self-reported extent of Facebook use (*r* = 0.38, *p* < 0.01) adapted from Turel (2015). These scales are presented in Appendix A in Supplementary Materials.

The main study's first wave survey included the following multi-item measures, all of which presented good reliability: (1) ADHD (Kessler et al., 2005, Part A of the ADHD-ASRS Screener v1.1, α = 0.72), (2) self-esteem (Rosenberg, 1965, α = 0.87), (3) social desirability (Reynolds, 1982, Short form of the Marlowe-Crowne social desirability scale. α is not reported since an index score is calculated), and (4) Facebook use habit (Verplanken and Orbell, 2003, Self-Report Index of habit strength applied to the case of Facebook use, α = 0.94). Note that the ASRS v1.1 encompasses questions which reflect DSM-IV-TR criteria (American Psychiatric Association, 2000). Part A includes six questions which are best associated with ADHD symptoms, and hence represents a valid short version of the full ASRS v1.1 scale and which can be used for initial ADHD screening (WHO, 2003). The first wave survey also captured age, sex (Male = 0, Female = 1), years of experience on the target SNS and number of target SNS friends, for descriptive and control purposes.

The main study's second wave survey included the following multi-item measures, all of which presented good reliability: (1) stress (Cohen et al., 1983, Perceived Stress Scale-Short, PSS-4, α = 0.90), and (2) craving to use the target SNS based on the Elaborated Intrusion (EI) theory of desire (May et al., 2004) using the FaCE questionnaire (based on Statham et al., 2011). The sub-scales were reliable with Cronbach's α scores of 0.93, 0.91, 0.92, 0.93, 0.90, and 0.90 for FaCE-S-imagery, FaCE-S-intensity, FaCE-S-intrusion, FaCE-F-imagery, FaCE-F-intensity, and FaCE-F-intrusion, respectively. The second wave survey also captured self-reported use of the target SNS while driving, using a single item based on the frequency of use measure by Turel (2015). These measures and items are presented in Appendix A Supplementary Materials.

### Data analysis

Descriptive statistics and correlations were calculated with SPSS 23. The confirmatory factor analysis model and the structural model were then estimated with the Structural Equation Modeling (SEM) facilities of AMOS 23 following the two-step approach (Anderson and Gerbing, 1988) and using common cut-off criteria for fit indices (Hu and Bentler, 1999). *Post-hoc* mediation tests were performed using the bootstrapping procedure by Preacher et al. (2007) using AMOS 23. Bootstrapping procedures are advantageous for mediation testing, since the product of two coefficients is not normally distributed (Cheung and Lau, 2008). Lastly, group comparison (having symptoms highly consistent with ADHD or not) was performed using multivariate analysis of variance techniques (MANOVA) with SPSS 23. This approach is an extension of the ANOVA model to situations in which multiple comparisons are to be conducted, i.e., there are multiple dependent variables (Pedhazur and Pedhazur Schmelkin, 1991). In such cases MANOVA is advantageous, since testing multiple ANOVA models biases type-I error and can lead to incorrect inferences (Tabachnick and Fidell, 2012). In addition, *post-hoc* sex moderation was examined using parameter pairwise comparisons in AMOS 23, comparing path-by-path between men and women.

## Results

### Model estimation

First, descriptive statistics for the model's constructs (including control variables) as well as correlations among them were calculated. These are given in Table 2 (control variables on the bottom). The table reveals that the correlations are in the expected directions. It further indicates women in our sample (coded as 1) felt higher levels of stress and had lower self-esteem; and perhaps consequently felt slightly stronger cravings to use the target SNS compared to men. Younger people had more contacts on the target SNS and a stronger SNS use habit compared to older people in our sample. Social desirability, as expected, was associated with reduced self-reports of negative phenomena, such as ADHD, stress, craving, and target SNS use while driving. It increased self-reports of positive phenomena such as self-esteem. Hence, it was concluded that it is important to control for it.

**Table 2:** **Descriptive Statistics and Correlations**.

**Construct**	**Mean (SD)**	**(1)**	**(2)**	**(3)**	**(4)**	**(5)**	**(6)**	**(7)**	**(8)**	**(9)**	**(10)**
(1) ADHD	2.79 (0.61)	−									
(2) Stress	2.61 (0.92)	0.43^**^	−								
(3) Self- Esteem	3.77 (0.72)	−0.26^**^	−0.36^**^	−							
(4) SNS Craving	7.17 (6.86)	0.26^**^	0.31^**^	−0.30^**^	−						
(5) SNS use While Driving	2.04 (1.60)	0.19^**^	0.15^^**^^	−0.07	0.33^^**^^	−					
(6) Age	23.4 (4.18)	−0.06	−0.09	0.01	−0.02	−0.04	−				
(7) Sex	−	−0.02	0.15^**^	−0.13^^**^^	0.10^*^	0.00	−0.05	−			
(8) Years on SNS	4.18 (2.23)	0.00	−0.01	0.00	0.05	−0.06	0.07	−0.02	−		
(9) Contacts on SNS	339.87 (441.98)	0.06	0.05	0.08	0.10^*^	0.14^**^	−0.16^**^	0.01	0.07	−	
(10) SNS Use Habit	4.47 (1.35)	0.21^**^	0.26^**^	−0.09	0.39^**^	0.29^**^	−0.22^**^	0.19^**^	0.05	0.24^**^	−
(11) Social Desirability	6.67 (2.77)	−0.32^**^	−0.33^**^	0.33^**^	−0.14^**^	−0.13^**^	0.02	−0.00	−0.03	0.02	−0.17^**^

* p < 0.05;

** p < 0.01.

**Figure 1:**
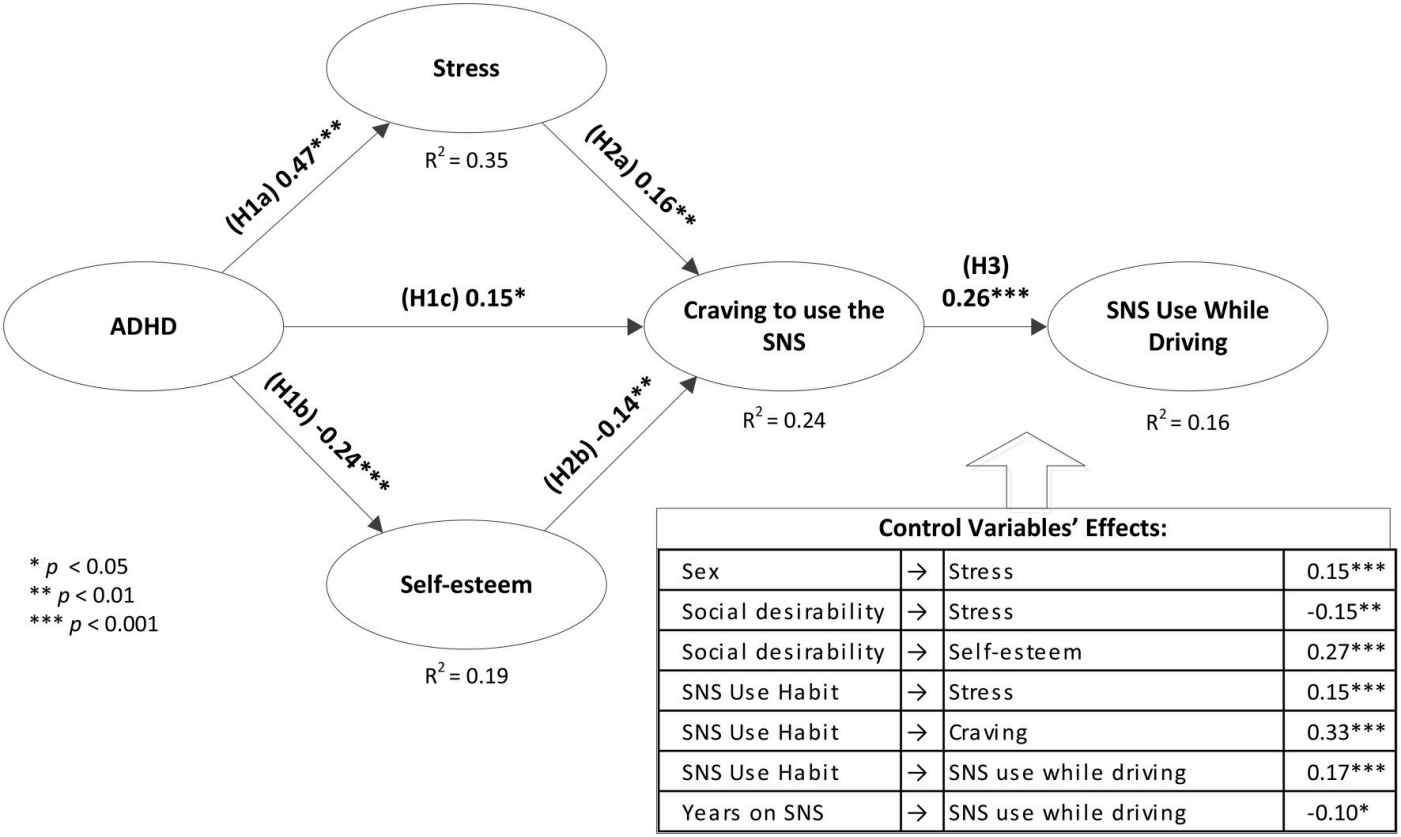
**Structural Model**.

Second, a confirmatory factor analysis (CFA) model was estimated with the multiple item constructs: ADHD, self-esteem, and stress and components of the FaCE scale. It presented good fit: χ^2^/df = 2.40, CFI = 0.95, IFI = 0.95, GFI = 0.93, RMSEA = 0.056, and SRMR = 0.066. Therefore, the structural model was estimated. In this model, ADHD, stress and self-esteem were modeled as latent factors and craving was modeled with an index which was calculated based on the procedure described in Statham et al. (2011). The model also accounted for possible effects of six control variables: age, sex, social desirability, habit, years on the target SNS, and contacts on the target SNS. The model presented good fit: χ^2^/df = 2.13, CFI = 0.93, IFI = 0.93, GFI = 0.91, RMSEA = 0.050, and SRMR = 0.061. Nevertheless, two control variables had no significant effects and hence, for parsimony reasons, were removed. The model was re-estimated, and still presented good fit: χ^2^/df = 2.19, CFI = 0.93, IFI = 0.93, GFI = 0.91, RMSEA = 0.051, and SRMR = 0.063. The standardized path coefficients, their levels of significance, and proportion of variances explained in endogenous constructs are provided in Figure 1.

### *Post-hoc* analyses

First, the proposed model implies a two-step partial mediation of the effect of ADHD on SNS use while driving, through stress, self-esteem and then through cravings. In order to examine these indirect effects, we employed the bootstrapping procedure described in Preacher et al. (2007) with 200 re-samples. Using this technique, the standardized bias-corrected indirect effects of ADHD on craving and SNS use while driving were 0.25 (*p* < 0.01) and 0.07 (*p* < 0.01), respectively. This further validates the proposed two-step indirect effect of ADHD on Facebook use while driving.

**Figure 2:**
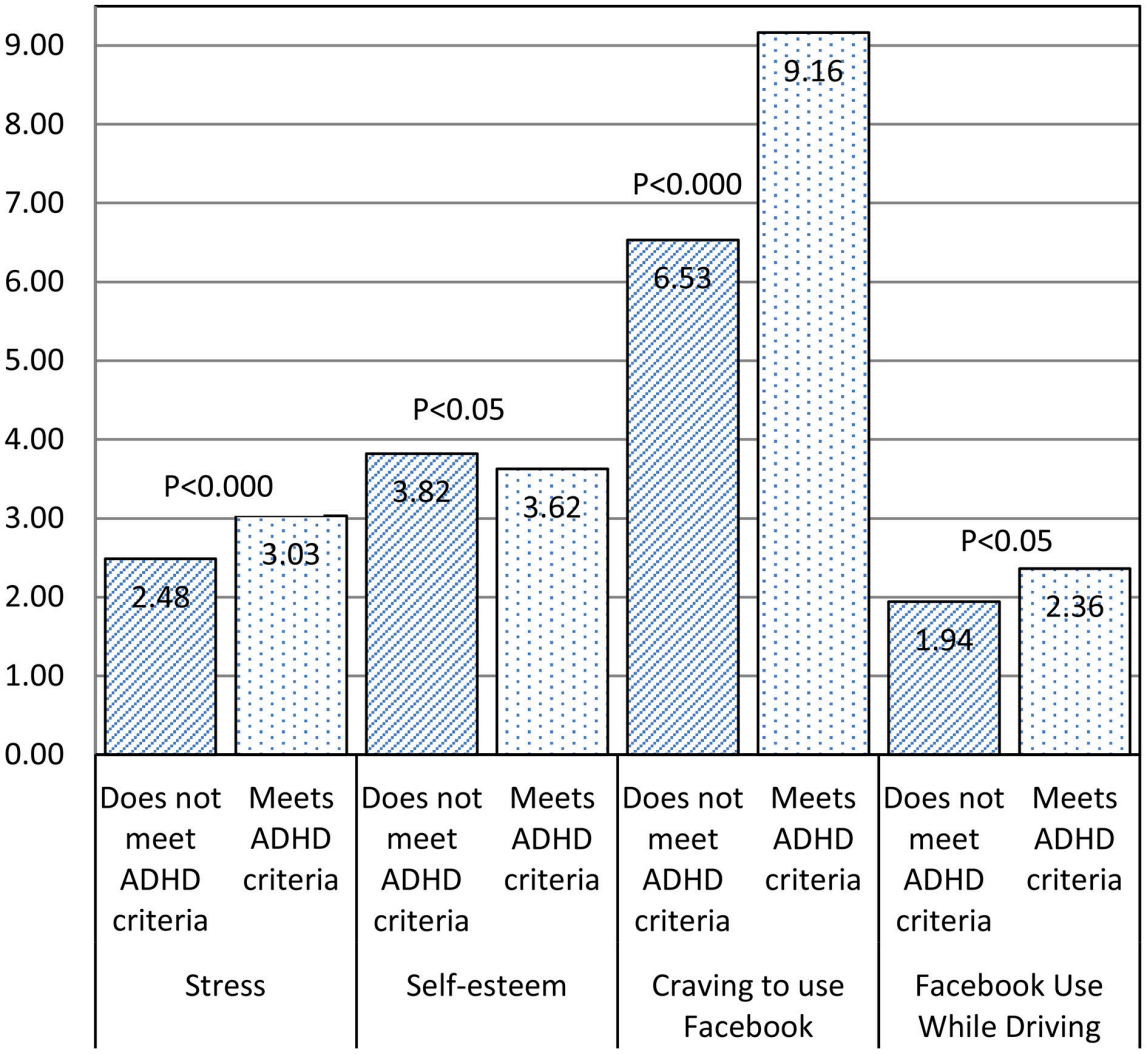
**Between-group differences**.

Second, using the ASRS v1.1 guidelines for scoring Part A (Kessler et al., 2005), individuals were classified as having symptoms highly consistent with ADHD (at least four symptoms above the specified thresholds; *n* = 110, 24%) or not (less than four symptoms over the specified threshold, *n* = 347, 76%). This binary variable represents a rough initial clinical assessment of potential ADHD (WHO, 2003) that should be further explored. This initial classification was then used as a fixed factor in a multivariate analysis of variance model with stress, self-esteem, craving, and target SNS use while driving as dependent variables. Results show that there are omnibus differences between the groups (Pilai's Trace of 0.08, *F*_(4, 452)_ = 9.2, *p* < 0.000). The differences between groups for each variable were also significant (See group means and levels of significance of the between-group differences in Figure 2).

Third, while the proposed model controlled for sex effects, it did not consider the possibility that the processes through which ADHD influences SNS use while driving may differ between the sexes. Such differences may be reasonable given that the outcomes of and behavioral responses to ADHD differ between the sexes among children (Gaub and Carlson, 1997) and adults (Ramos-Quiroga et al., 2013). In addition, the sexes can differ in decision making processes under stress (Lighthall et al., 2012). In order to shed light on these differences, parameter pairwise comparisons were generated in AMOS 23. The unstandardized coefficients for which there was a significant difference, the z-scores for the differences, and the *p*-values are given in Table 3. Cravings to use the SNS and SNS use habit had stronger effects on SNS use while driving for men than for women. The resultant behavior seemed to be socially undesirable only for women.

**Table 3:** **Differences in Path Coefficients between the Sexes**.

			**Women**		**Men**		
			**Estimate**	***P***	**Estimate**	***P***	**z-stat**
SNS use while driving	< —	Craving to use the SNS	0.04	0.01	0.09	0.00	−2.38^**^
SNS use while driving	< —	Social Desirability	−0.08	0.03	0.01	0.78	−1.744^*^
SNS use while driving	< —	SNS use Habit	0.11	0.19	0.30	0.00	−1.732^*^

** p < 0.05;

* p < 0.10.

## Discussion

Can ADHD symptoms be an indirect factor contributing to SNS use while driving? And if so, can ADHD be viewed as an underlying syndrome that promotes this behavior, perhaps in a manner that is similar to how an addictive syndrome promotes compulsive seeking of drug rewards? This study sought to address these questions and the results points to several contributions.

The findings based on a two-wave survey of users of a popular SNS who drive to work/school lend support to these views. They show that the severity of ADHD symptoms is positively associated with SNS use while driving. There are also significant differences between the self-reported SNS use while driving behaviors of those who have symptoms highly consistent with ADHD and those who do not. The *post-hoc* analysis further supports this idea and demonstrates using bootstrapping and SEM techniques that the bias-corrected indirect effects of ADHD on SNS use while driving was significant. This indirect effect was partially mediated through the increased stress and reduced self-esteem ADHD symptoms had promoted (H1a and b were supported), which together with ADHD symptoms exacerbated cravings to use the SNS (H2a, b and c were supported). The elevated levels of cravings directly drove SNS use while driving, lending support to H3.

The first contribution of this study is in pointing to an important yet unexplored risky behavior associated with ADHD symptoms, namely SNS use while driving. Thus far, research has primarily focused on a family of risky behaviors associated with ADHD, which includes deviant work and interpersonal behaviors, gambling and substance use behaviors (Groen et al., 2013; Furukawa et al., 2014; Kirino et al., 2015). These behaviors can certainly be problematic and have shown to lead to various adverse consequences for adults (Wender et al., 2001; Okie, 2006; Davidson, 2008), including an increased risk of road accidents (Barkley et al., 1993). Our findings show that SNS use while driving is not only prevalent among the general user population (more than 40% of respondents in our sample engaged in this behavior during a week period, and a single digit percent engaged in it quite frequently), but also that this behavior is more prevalent among people who have symptoms highly consistent with ADHD and that this behavior is associated, indirectly, with the level of ADHD symptoms one presents.

These results first suggest that SNS use while driving may be more prevalent than previously assumed (the RAC report from 2011 claimed that in the UK 24% of 17–24 year olds and 12% of 25–44 year olds used SNS, email or other SNS while driving, RAC, 2011). Hence, the phenomenon of using SNS while driving in general, and especially among people with some underlying brain dysfunction in decision-making systems, such as ADHD, merits more attention and further research.

This need is exacerbated by the fact that the use of popular SNS can be highly tempting and rewarding, since it has the potential to alleviate negative feelings, social shortcomings, and other psychological burdens (Ryan and Xenos, 2011; Sheldon et al., 2011). The problem with such sites is that, as opposed to other means (e.g., alcohol, cannabis) which can be used for alleviating ADHD-related burdens, it is generally more accessible (at least in the United States wireless data plans are almost, if not fully, unlimited), cheaper, and worst of all- can be used spontaneously while driving, without much planning. Indeed, many people respond faster to SNS cues than to street signs (Turel et al., 2014), and many others use SNS while driving (Burns, 2015). Thus, cellphone users with data plans are driving around with a “loaded gun,” which can be easily triggered by SNS use cues (Turel et al., 2014). If we further take into account the increase in prevalence of ADHD symptoms in adults (Kessler et al., 2006; Fayyad et al., 2007; Simon et al., 2009), this study points to a greater need to study how ADHD and SNS use while driving are related, and how this association can be weakened or prevented.

The second contribution of this study is in conceptually tying ADHD with neuro-behavioral models of addiction as a means to partially explain impulsive and risky behaviors among ADHD sufferers. Contemporary theories of addiction have suggested that abnormalities in at least three different neural systems could facilitate compulsive seeking of drug reward: One is a dysfunctional prefrontal system involved in decision-making and impulse control; a second is a dysfunctional mesolimbic dopamine/striatal system involved in reward seeking and impulsivity; a third is a dysfunctional interoceptive system that includes the insula. This system becomes engaged by physiological need and homeostatic imbalance, such as what occurs during withdrawal, stress, and anxiety, and which ultimately result in craving and compulsive urge to seek relief or alleviation of the aversive state (Noel et al., 2013). Since ADHD may impact these neural systems in a similar way (e.g., ADHD often involves hypo-active inhibition systems and/or hyper-active impulsive brain systems), we propose that ADHD symptoms can promote reward seeking behaviors or aversive state alleviating behaviors such as SNS use while driving. Thus, SNS use while driving can be, in part, used as a means to alleviate one's cravings as influenced by burdens stemming from the not-easy to deal with core ADHD symptoms (Sousa et al., 2011; Silva et al., 2014).

This behavior can also be conceived as driven by incentive reward which is failed to be inhibited when dysfunctions in key brain systems, such as a hypoactive prefrontal cortex system for inhibitory control, and/or a hyperactive amygdala-striatal system for reward seeking and impulsive risk taking are present (Bechara et al., 1999, 2006; Noel et al., 2013). Engagement of the insula system by the cravings indicated earlier exacerbates the hypo-activity of the impulse control system and the hyperactivity of the system that drives impulsive behaviors (Bechara et al., 1999, 2006; Noel et al., 2013). The findings of this study lend initial support to these views and demonstrate that ADHD symptoms drive adverse states including reduced self-esteem and increased stress, and that these factors together increase one's cravings to use SNS. These cravings, in turn, when fail to be inhibited, translate into impulsive behaviors. Given the underlying similarities between ADHD and other syndromes entailing weaknesses in decision making abilities, such as addiction disorders (Malloy-Diniz et al., 2007), the findings point to the possibility that SNS use while driving can be rooted in problems related to the same brain regions. The roles of these neural mechanisms in promoting this specific behavior, though, require further research using brain imaging techniques.

The third contribution of this study is in pointing to processes which might mediate the effects of ADHD symptoms on SNS use while driving. This focus is important since tackling the mediating variables can help in reducing the problematic (and largely illegal and prohibited, at least in the US) behavior; and in essence prevent the translation of ADHD symptoms into this behavior. Specifically, our findings imply that the use of SNS while driving can be reduced through decreasing one's cravings to use the target SNS and his or her stress, while increasing his or her self-esteem. While we did not test techniques for achieving these changes, prior research implies that such changes may be attained through behavioral therapy interventions (Knapen et al., 2005), lifestyle changes (Sundin et al., 2003), and the possible use of pharmacology and other non-invasive techniques such as transcranial magnetic stimulation (Forget et al., 2010) in more severe cases. The efficacy of such approaches for reducing SNS use while driving, though, should be examined in future research.

The fourth contribution of this study is in expanding the body of knowledge regarding sex differences related to ADHD and SNS use while driving. While prior research has pointed to such differences related to risky behaviors such as substance abuse, response to stress, and decision making (Gaub and Carlson, 1997; Lighthall et al., 2012; Willis and Naidoo, 2014), the way one's sex may influence the way SNS use while driving behaviors are formed is still unknown. Our findings (see Table 3) indicate that cravings to use SNS are more potent among men. Hence, intervention strategies may first target males. They also indicate that for men, using SNS while driving is not negatively or positively associated with social desirability and for women, lower levels are more socially desirable. This again, may require corrective action among males. Lastly, the habituation of SNS use seems to be a stronger driver for SNS use while driving for men than for women. This implies that habit correction interventions can better help men as an indirect means for reducing SNS use while driving. Such sex-based intervention approaches, though, should be examined in future research.

Some limitations and future research directions should be acknowledged. First, this study employed only one part of the ASRS and hence ADHD diagnostics could not be obtained. However, having symptoms consistent with ADHD was sufficient to show differences among people in terms of SNS use while driving. Second, the study focused on only a few variables which mediate the association between ADHD symptoms and SNS use while driving. While we correctly assumed that these are viable mediators, there are possibly many others; and these should be explored in future research. In addition, the risk of SNS use while driving may differ based on the activity (checking vs. updating) and the traffic conditions. Such variables may be accounted for in future research. Third, while we imply association of the examined processes to brain systems involved in impulsions, cravings and inhibition, these were not tested. We hence call for future research to use additional techniques, such as fMRI, to corroborate our findings, and add a brain functioning layer to our understanding of the association between the examined constructs. Lastly, this study has focused on one instance of SNS, Facebook. While Facebook is perhaps the most popular SNS, there are many other SNS which can be presumably also used while driving. Future research should examine our model with other SNSs and/or risky and rewarding behaviors in order to increase its generalizability.

## Conclusion

ADHD and addictive syndromes are rooted in deficits in similar brain systems involved in impulsion generation and control. In this study we showed that consequently, risky behaviors among people with symptoms consistent with ADHD can be explained using an addictive symptom perspective. We also demonstrated that SNS use while driving is a growing problem in society, and that it is more prevalent among people with symptoms consistent with ADHD. Future research should further study these phenomena and explore ways for reducing risky technology use while driving.

## Author contributions

The first author (OT) was involved in study design, implementation, execution, data analysis and write-up. The second author (AB) was involved in study design, theorizing and write-up.

### Conflict of interest statement

The authors declare that the research was conducted in the absence of any commercial or financial relationships that could be construed as a potential conflict of interest.
