# Supplementary material for: Social Networking Site Use While Driving: ADHD and the Mediating Roles of Stress, Self-Esteem and Craving
Source: Front Psychol. 2016 Mar 30;7:455. doi: 10.3389/fpsyg.2016.00455 (PMC4812103; doi:10.3389/fpsyg.2016.00455)
Supplement: Supplementary file 1 [file DataSheet1.pdf]

APPENDIX A- MEASURES

Pilot Study (t_0)

SNS Cravings in the case of Facebook:

Facebook Craving Experience (FaCE) scale

Please reflect on the times last week you had no access to this social networking site (Facebook) and describe your general experience of desire to use the site. When not able to access this site....

<u>Dimension 1: Facebook Craving Experience– Strength (FaCE-S)</u>

<u>Component 1:</u> Imagery [1=Very vaguely, 7=Very vividly]

- How vividly did you picture the use of the site?
- How vividly did you imagine what it would feel like to use the site?
- How vividly did you imagine how relieved you would feel if you had used the site?

<u>Component 2:</u> Intensity [1=Not at all/ Very weak, 7=Very strongly/ Very hard]

- How strongly did you want to use of the site?
- How much did you feel you needed to use the site?
- How strong was the urge to use the site?
- How hard was it to think about anything else?

<u>Component 3:</u> Intrusion [1=Very vaguely, 7=Very vividly]

- How hard were you trying not to think about this site?
- How intrusive were the thoughts about this site?

<u>Dimension 2: Facebook Craving Experience– Frequency (FaCE-F)</u>

<u>Component 1:</u> Imagery [1=Never, 7=Very often]

- How often did you picture the use of the site?
- How often did you imagine what it would feel like to use the site?
- How often did you imagine how relieved you would feel if you had used the site?

<u>Component 2:</u> Intensity [1=Never, 7=Very often]

- How often did you want to use of the site?
- How often did you feel you needed to use the site?
- How often did you have an urge to use the site?
- How often did you find it hard to think about anything else?

<u>Component 3:</u> Intrusion *[1=Never, 7=Very often]*

- How often were you trying not to think about the site?
- How often were the thoughts about this site intrusive?

Urge to use SNS in the case of Facebook

Recall or imagine a situation in which you had/have no Facebook access for a week (e.g., when you are/were on a trip to a place with no Internet access). How would you feel immediacy after your return, when you turn on your cellphone, tablet or PC for the first time:

[1=Strongly disagree, 7=Strongly agree]

- All I will want to do when I return is to use my social networking website.
- It would be difficult to turn down the opportunity to use my social networking website when I return.
- Using my social networking website after my return would make things seem just perfect.
- I will want to use my social networking website so bad when I return that I can almost feel it.
- Nothing would be better than using my social networking website when I return.
- I will crave using my social networking website when I return.

SNS Temptation and Restraint Inventory applied to Facebook:

Facebook Temptation and Restraint Inventory (F-TRI)

Dimension 1: Cognitive-emotional preoccupation with Facebook (CEP-F)

Component 1: Emotion [1=Never, 7=Very often]

Reflecting on your Facebook use over the last week - How often...

- ...when you felt anxious, you craved to use the site?
- ...when you felt lonely, you felt an urge to use the site?
- ...did you feel so nervous that you really needed to use the site?

Component 2: Cognitive Preoccupation [1=Never, 7=Very often]

Reflecting on your Facebook use over the last week - How often...

- ...did you find yourself unable to stop thinking about using the site?
- ...was it hard to distract yourself from thinking about your social networking site?
- ...did thoughts about using your site intrude into your daily activities?

Component 3: Govern [1=Very little, 7=Very high/a lot]

Reflecting on your Facebook use over the last week - Please answer the following questions:

- How much effort did it take for you to keep your use under control?
- How much difficulty did you have controlling your use?
- How difficult was it for you to stop using the site once you start using it?

Dimension 2: Cognitive-behavioral control efforts regarding Facebook (CBC-F)

Component 1: Concern [1=Never, 7=Very often]

Reflecting on your Facebook use over the last week - How often...

- ...did the sight of your site made you think about limiting your use of it?
- ...did seeing the site stimulate concerns about the need to limit your use?
- ...did seeing other people using a social networking site remind you of your efforts to control your use?

Component 2: Restrict [1=Never, 7=Very often]

Reflecting on your Facebook use over the last week - How often...

- ...did you find that once you start using this site it was difficult for you to stop?
- ...did you cut back on using this site in an attempt to restrain your use habits?
- ...did feelings of guilt about using the site too much help you to control your use?

Extent of SNS (Facebook) use

Overall, how do you consider the extent of your Facebook use over the [previous week? [1=Very light use, 5=Very heavy use]

Main Study- First Wave (t_1)

ADHD (© World Health Organization 2003)

Please answer the questions below, rating yourself on each of the criteria shown using the scale on the right side of the page. As you answer each question, please select the answer that best describes how you have felt and conducted yourself over the past 6 months.

[1=Never, 5=Very often]

- How often did you have trouble wrapping up the final details of a project, once the challenging parts have been done?
- How often did you have difficulty getting things in order when you have to do a task that requires organization?
- How often did you have problems remembering appointments or obligations?
- When you had a task that required a lot of thought, how often did you avoid or delay getting started?
- How often did you fidget or squirm with your hands or feet when you had to sit down for a long time?
- How often did you feel overly active and compelled to do things, like you were driven by a motor?

Self-esteem

Reflecting on the way you see yourself (and not how you would like to be), please rate how much you agree with each one of these statements [1=Strongly disagree, 5=Strongly agree]:

- I feel that I am a person of worth, at least on an equal plane with others.
- I feel that I have a number of good qualities.
- All in all, I am inclined to feel that I am a failure.
- I am able to do things as well as most other people.
- I feel I do not have much to be proud of.
- I take a positive attitude toward myself.
- On the whole, I am satisfied with myself.
- I wish I could have more respect for myself.
- I certainly feel useless at times.
- At times I think I am no good at all.

Social Desirability

Please indicate whether the statements below are true or false with respect to yourself. No two statements are exactly alike, so consider each statement carefully before answering. If a statement is TRUE or MOSTLY TRUE as applied to you, pick "True." If a statement is FALSE or MOSTLY FALSE as applied to you, pick "False." [True or False]

- It is sometimes hard for me to go on with my work if I am not encouraged.
- I sometimes feel resentful when I don't get my way.
- On a few occasions, I have given up doing something because I thought too little of my ability.
- There have been times when I felt like rebelling against people in authority even though I knew they were right.
- No matter who I'm talking to, I'm always a good listener.
- There have been occasions when I took advantage of someone.
- I'm always willing to admit it when I make a mistake.
- I sometimes try to get even, rather than forgive and forget.
- I am always courteous, even to people who are disagreeable.
- I have never been irked when people expressed ideas very different from my own.
- There have been times when I was quite jealous of the good fortune of others.
- I am sometimes irritated by people who ask favors of me.
- I have never deliberately said something that hurt someone's feelings.

SNS (Facebook) Use Habit

Using Facebook is something….

- I do frequently.
- I do automatically.
- I do without having to consciously remember.
- that makes me feel weird if I do not do it.
- I do without thinking.
- that would require effort not to do it.
- that belongs to my daily routine.
- I start doing before I realize I'm doing it.
- I would find hard not to do.
- I have no need to think about doing.
- that's typically "me."
- I have been doing for a long time.

Other controls/ descriptive variables

What is your **age**?

What is your **sex**? [0=Male, 1=Female]

How many ***years ago*** did you start using this social networking site?

How many ***contacts*** do you have in this social networking site?

Main Study- Second Wave (t_2)

Stress

Please reflect on your life situation over the last week, and state how often you have felt like the statements below. In the LAST WEEK...[1=Never, 5=Very often]

- ...how often have you felt that you were unable to control the important things in your life?
- ...how often have you felt UNconfident about your ability to handle your personal problems?
- ...how often have you felt that things were NOT going your way?
- ...how often have you felt difficulties were piling up so high that you could not overcome them?

Facebook Craving Experience (FaCE) scale

Same as in pilot study.

SNS (Facebook) Use While Driving

- Over the PREVIOUS WEEK, how often did you use the website while driving (even just checking)? [1= Never/Very rarely, 7=Very often]
